# Ultra-High-Performance Liquid Chromatography–High-Definition Mass Spectrometry-Based Metabolomics to Reveal the Potential Anti-Arthritic Effects of *Illicium verum* in Cultured Fibroblast-like Synoviocytes Derived from Rheumatoid Arthritis

**DOI:** 10.3390/metabo14100517

**Published:** 2024-09-25

**Authors:** Mingzhen Qin, Lu Chen, Xiaoli Hou, Wuwei Wu, Yu Liu, Yu Pan, Mengli Zhang, Zhien Tan, Danna Huang

**Affiliations:** National Engineering Research Center of Southwest Endangered Medicinal Resources Development, Guangxi Botanical Garden of Medicinal Plants, 189 Changgang Road, Nanning 530023, Chinawuww@gxyyzwy.com (W.W.); tanze@gxyyzwy.com (Z.T.)

**Keywords:** rheumatoid arthritis, cell metabolomics, *Illicium verum*, fibroblast-like synoviocytes, ultra-high-performance liquid chromatography–high-definition mass spectrometry

## Abstract

Background: Rheumatoid arthritis (RA) is a chronic inflammatory autoimmune disease. The fruits of *Illicium verum*, which is a medicinal and edible resource, have been shown to have anti-inflammatory properties. Methods: In this study, we investigated the effects of *I. verum* extracts (IVEs) on human RA fibroblasts-like synoviocytes (RA-FLS) by using a sensitive and selective ultra-high-performance liquid chromatography with high-definition mass spectrometry (UPLC-HDMS) method. We subsequently analyzed the metabolites produced after the incubation of cultured RA-FLS with IVEs. Results: IVEs inhibited the proliferation and suppressed the migration of RA-FLS, and reduced the levels of inflammatory factors including TNF-α and IL-6. Twenty differential metabolites responsible for the effects of IVEs were screened and annotated based on the UPLC-HDMS data by using a cell metabolomics approach. Discussion: Our findings suggest that treating RA-FLS with IVEs can regulate lipid and amino acid metabolism, indicating that this extract has the potential to modify the metabolic pathways that cause inflammation in RA. Conclusions: This might lead to novel therapeutic strategies for managing patients with RA.

## 1. Introduction

Rheumatoid arthritis (RA) is a common systemic autoimmune disease [1]. Inflammation in autoimmune rheumatic diseases such as RA involves various immune cell subpopulations with distinct metabolic needs, such as chronic T cell mitochondrial hyperpolarization caused by hypoxia in RA synovial tissues [2]. Cytokine inhibitors have been proven to play a critical role in the pathogenesis of diseases, particularly by reducing the levels of tumor necrosis factor α (TNF-α) and interleukin 6 (IL-6) [3]. Current RA therapies primarily focus on managing symptoms and reducing inflammation through the use of nonsteroidal anti-inflammatory drugs (NSAIDs), disease-modifying anti-rheumatic drugs (DMARDs), and biologic response modifiers [4,5]. Nevertheless, drugs administered through RA therapy come with a range of side effects, including gastrointestinal damage, hepatorenal toxicity, and allergic reactions, and patients often have poor adherence to them [6,7,8]. Therefore, there is a heightened focus on developing new drugs, which can elicit fewer adverse reactions and exhibit lower toxicities.

*Illicium verum*, commonly known as Chinese star anise, holds a significant position within the domain of traditional Chinese medicine and spices. It possesses a multitude of beneficial properties, such as antioxidant, antibacterial, fungicidal, anti-inflammatory, anesthetic, and anti-nociceptive effects, and it has a unique and distinctive flavor due to the presence of essential oils [9]. *I. verum* has been demonstrated to alleviate rheumatism and joint pain, repel insects, reduce cold and flu symptoms, and can act as an antioxidant by combating oxidative stress, inhibiting cell death, preventing DNA damage and scavenging free radicals [10]. The potential clinical applications of *I. verum* in RA treatment include its ability to modulate the immune response and reduce joint inflammation. By constructing experimental animal models of hot plate analgesia, yeast-induced fever, and plantar edema, it has been observed that the extracts derived from the dried fruits of *I. verum* exhibited significant analgesic, antipyretic, and anti-inflammatory effects at different doses in a dose-dependent manner with varying potencies [11]. The essential oil derived from *I. verum* is applied in rheumatism, as recommended by some folk remedies [12]. It might be a useful intervention to reduce inflammation and joint damage, and it is associated with the down-regulation of cytokines and nitric oxide levels [13].

Fibroblast-like synoviocytes (FLS) are integral cellular elements residing in the internal layer of the joint capsules. However, during inflammatory processes, such as in RA, they may assume a pivotal role in perpetuating inflammation due to their destructive capabilities [14,15]. RA-FLS displays an aggressive tumor-like behavior, characterized by resistance to apoptosis, growth without attachment, loss of contact inhibition, and oligoclonal expansion. This hyperplastic synovial tissue may result from either faster proliferation or slower apoptosis of FLS [16]. Consequently, the exploration of potential pharmaceutical interventions targeting RA-FLS has emerged as a promising approach for managing patients with RA.

Cellular metabolomics involves the specific metabolites in cells, and it is the best indicator to characterize the phenotype of organisms, while metabolomics is a tool for the systematic analysis of cellular metabolic fingerprints and has been widely used to study cellular metabolism [17]. Therefore, in this study, we will describe the effects of *I. verum* on the metabolic fingerprint of RA-FLS to analyze the changes in cellular metabolic pathways. We also aimed to elucidate the mechanisms of *I. verum* in the treatment of RA. This will provide a theoretical basis for the discovery of anti-RA drugs with significant clinical effects but relatively few side effects.

## 2. Materials and Methods

### 2.1. Chemicals and Reagents

LC/MS grade acetonitrile and methanol were supplied by ThermoFishier (Waltham, MA, USA). Analytical grade formic acid was obtained from Aladdin (Shanghai, China). Ultra-pure water was purchased from Watsons (Guangzhou, China), and dimethyl sulfoxide (DMSO) was from Solarbio (Beijing, China). Fetal bovine serum (FBS) and Dulbecco’s modified Eagle medium (DMEM) with high glucose was obtained from Gibco (Grand Island, NE, USA). The Cell Counting Kit-8 (CCK-8) was purchased from Biosharp (Hefei, China). Enzyme-linked immunosorbent assay (ELISA) kits for TNF-α and IL-6 were purchased from Bioswap (Wuhan, China). An immortalized fibroblast-like synoviocyte cell lines derived from synovial tissue of patients with RA was purchased from Anwei-sci (Shanghai, China).

### 2.2. Herbal Extraction Preparation

The dried fruits of *I. verum* were collected from Shanglin County in Guangxi. Professor Xiaolei Zhou from the Guangxi Key Laboratory of Medicinal Resources Protection and Genetic Improvement identified the samples of this species, and it was judged to reach the standards stipulated in the Chinese Pharmacopoeia by Guangxi Chinese Medicinal Materials Product Quality Supervision and Inspection Station. A voucher specimen (No.2220904SL_IV/F) was deposited in the National Engineering Research Center of Southwest Endangered Medicinal Resources Development, Guangxi Botanical Garden of Medicinal Plants.

The ethanol extract of *I. verum* was obtained as previously reported [11]. The dried and powdered fruits of *I. verum* (560 g) were extracted three times with 75% ethanol (6 L × 3) under reflux conditions at 70 °C, and each extraction period lasted for 1.5 h. The extracts were filtered through a Brinell’s funnel, and then they were combined and subsequently evaporated via a rotary vacuum evaporator. Finally, the concentrates were lyophilized using a vacuum freeze-dryer to obtain an ethanol extract of *I. verum* with a yield of 134.3 g. The *I. verum* extracts (IVEs) were dissolved in DMSO to obtain a stock solution. This stock solution was diluted as necessary with fresh DMEM culture medium before use.

### 2.3. Cell Culture

RA-FLS were grown in DMEM containing 10% (*v*/*v*) FBS and 1% (*v*/*v*) antibiotic-antimycotic solution. All cells were incubated in an atmosphere of 5% CO_2_ at 37 °C.

### 2.4. Cell Proliferation Assay

In total, 5 × 10^3^ cells per well were seeded in 96-well plates and cultured for 24 h. Subsequently, the cells were, respectively, treated with IVEs at various concentrations of 1.5, 3, 6, 12, 25, and 50 μg/mL or with vehicle only as control. After incubation for 24, 48, and 72 h, respectively, cell proliferation assays were conducted according to the manufacturer’s instructions for the CCK-8 assay, and the absorbance values were measured at 450 nm by using a microplate reader.

### 2.5. Cell Migration Assay

The effect of IVEs on RA-FLS cell migration was assessed using transwell chambers (Corning, New York, NY, USA). The cells were divided into four groups, including RA-FLS without IVEs as control and RA-FLS treated with IVEs at concentrations of 6, 12, and 25 μg/mL. In total, 5 × 10^4^ cells/well in serum-free medium together with various treatments were added to the upper chambers of transwells and DMEM with 10% FBS was added to the lower compartments. After 24 h, the non-migrated cells on the upper side of the chambers were wiped with a cotton swab and the migrated cells on the lower side of the chamber were fixed with methanol for 25 min. These were stained with 0.1% crystal violet. After rinsing with phosphate buffered saline (PBS), five fields of view were randomly selected using a microscope and the numbers of cell that migrated were counted.

### 2.6. Cell Inflammatory Factors Analysis

RA-FLS were treated with various concentrations of IVEs (6, 12, and 25 μg/mL) and then they were incubated for 48 h. After that, the supernatants were collected by centrifugation, and the levels of TNF-α and IL-6 were measured by ELISAs by using the corresponding commercially available kits.

### 2.7. Cell Metabolomic Analysis

#### 2.7.1. Sample Preparation

Cell metabolomic profiling of RA-FLS was performed with a modification to previous studies [18]. RA-FLS were seeded in 6-well plates (1 × 10^6^ cells/well) treated with IVEs at a concentration of 6 μg/mL with DMEM alone as control, respectively. After 48 h incubation, the cells were rinsed with pre-chilled PBS. In total, 1 mL of cold (−80 °C) aqueous methanol solution (80%) was added into each well to the remaining cells to quench the metabolism, and the intracellular metabolites were extracted. These were cooled immediately to −80 °C for 30 min and then they were stored. Subsequently, the cells were scraped and the extraction mixtures were transferred to 2 mL Eppendorf tubes. Then, samples were centrifuged at 15,000 rpm at 4 °C for 10 min. Following that, the supernatants were retained and then they were dried in a nitrogen atmosphere. The dried samples were reconstituted with 600 μL of 85% methanol and then centrifuged at 12,000 rpm under 4 °C for 15 min. The supernatants were filtered through 0.22 μm microfiltration membranes and then they were transferred to new tubes for metabolomics analysis. Equivalent mixing of each sample ensured quality control of samples.

#### 2.7.2. UPLC-MS Analysis

Chromatography was performed on the Acquity UPLC BEH C18 column (2.1 × 100 mm, 1.7 μm, Waters, Milford, MA, USA) using a Waters Acquity ultra-high performance liquid chromatography system (Waters, USA) coupled to a Waters SYNAPT G2-Si high-definition mass spectrometry system (Waters, USA) that was operated using MassLynx V4.2 software (Waters, USA). The gradient eluting system consisted of the mobile phase A containing 0.1% formic acid in water and B containing 0.1% formic acid in acetonitrile. The detailed gradient eluting conditions were: from 0 to 1 min, and from 98 to 96% A; from 1 to 1.5 min, and from 96 to 50% A; from 1.5 to 3 min, and from 50 to 35% A; from 3 to 5 min, and from 35 to 20% A; from 5 to 6 min, and from 20 to 16% A; from 6 to 7 min, and from 16 to 10% A; from 7 to 10 min, and from 10 to 2.5% A; from 10 to 11 min, and from 2.5 to 2% A; from 11 to 12 min, 2% A isocratic; from 12 to 13 min linear increase from 2 to 98% A; and 13–15 min, 98% A isocratic. The MS scan was operated in both the positive and negative ionization modes, and these ranged from 50 to 1500 Da.

#### 2.7.3. Data Processing

The raw UPLC-HDMS data of the samples were imported to Progenesis QI V2.0 software (Waters, USA) for chromatographic peak alignment, selection, and normalization. After data pre-processing, the metabolic data were analyzed using EZinfo 3.03.0 software to perform a principal component analysis (PCA) and orthogonal partial least squares discriminant analysis (OPLS-DA) for screening potential differential metabolites. Subsequently, the MassFragment™ manager (Waters, USA) was employed to identify the metabolites, and this was combined with a series of database including the Human Metabolome Database (HMDB) (http://www.hmdb.ca/, accessed on 7 September 2023), Chemspider (http://www.chemspider.com/, accessed on 7 September 2023), LIPID MAPS (https://lipidmaps.org, accessed on 7 September 2023), and National Metabolomics Data Repository (https://www.metabolomicsworkbench.org/, accessed on 7 September 2023), as well as comparing the mass spectra with those of ion fragments of certain compounds. The metabolic pathway network related to RA that were affected was assessed via the MetaboAnalyst 6.0 (http://www.metaboanalyst.ca/ accessed on 19 March 2024) and Kyoto Encyclopedia of Genes and Genomes (KEGG) (http://www.genome.jp/kegg/, accessed on 19 March 2024).

### 2.8. Statistical Analysis

The data were analyzed with GraphPad Prism version 8.3.0 software (GraphPad Software, Inc., San Diego, CA, USA) and they were expressed as means ± SDs. The differences between two groups were assessed using one-way analysis of variance (ANOVA). A *p*-value < 0.05 was considered statistically significant.

## 3. Results

### 3.1. IVEs Inhibited the Cell Proliferation of RA-FLS

**Figure 1:**
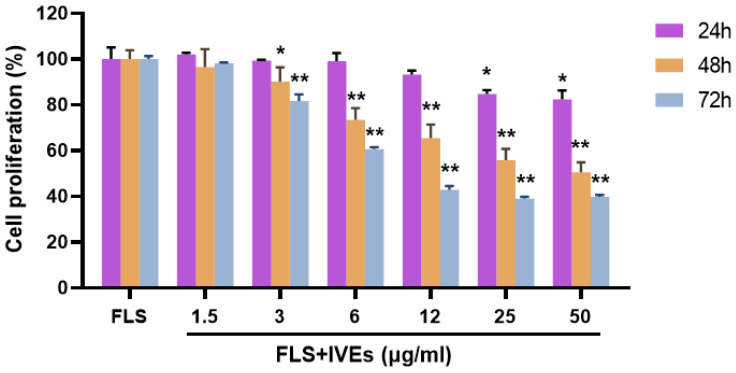
The effects of *Illicium verum* extracts on RA-FLS proliferation. The effect of different concentrations of IVEs on the percentage of RA-FLS proliferation at 24, 48, and 72 h, was assessed by using the CCK-8 assay. The data are expressed as means ± SDs (*n* = 3). * and ** represent *p* < 0.05 and 0.01 compared with the untreated group, respectively.

The effects of IVEs on the proliferation of RA-FLS were evaluated using the CCK-8 method. The RA-FLS cell survival rate decreased in a concentration and time-dependent manner (Figure 1). Compared with the FLS untreated group, 25 and 50 μg/mL IVEs significantly inhibited cell proliferation administration at 24 h (*p* < 0.05). At 48 and 72 h, the cell proliferation was also inhibited significantly (*p* < 0.01) when compared with the untreated group. Due to its inhibitory effect on cell proliferation and lower cytotoxicity, the 6 μg/mL IVEs treatment was selected for subsequent experiments.

### 3.2. IVEs Inhibited the Cell Migration of RA-FLS

**Figure 2:**
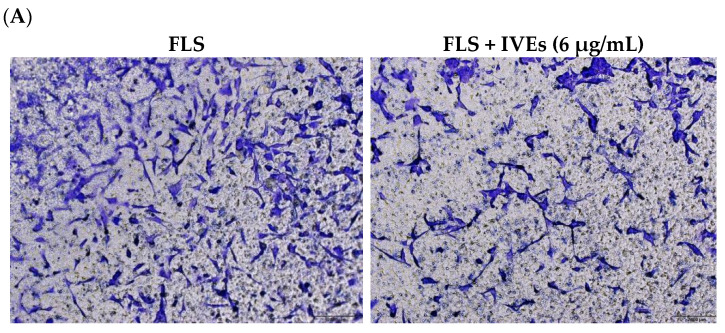

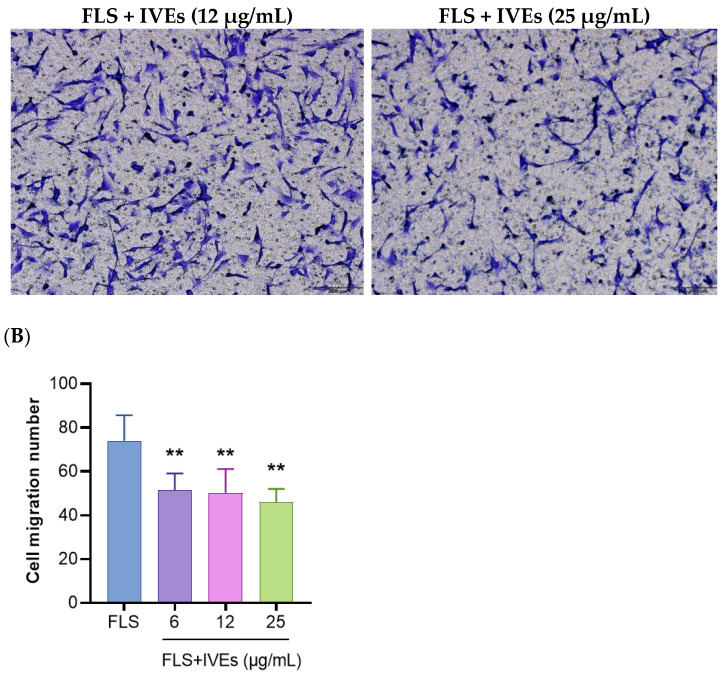
Effects of *Illicium verum* extract on RA-FLS migration. RA-FLS were seeded in the upper chambers of transwells with serum-free medium and these were treated with vehicle as control and IVEs (6, 12, and 25 μg/mL). The migrated RA-FLS were fixed, stained, photographed, and quantified. (**A**) Photograph of the migrated cells. The migrated RA-FLS in five randomly selected fields were counted and photographed under a light microscope (×100). (**B**) Quantification of cell migration. The data are expressed as means ± SDs (*n* = 5). ** represent *p* < 0.01 compared with untreated group.

The transwell migratory assay was performed on RA-FLS to investigate the effects of IVEs on RA. The cell migration capability of RA-FLS was significantly inhibited by IVEs (*p* < 0.05) when compared to the untreated group (Figure 2).

### 3.3. IVEs Inhibited the Production of Inflammatory Factors from RA-FLS

**Figure 3:**
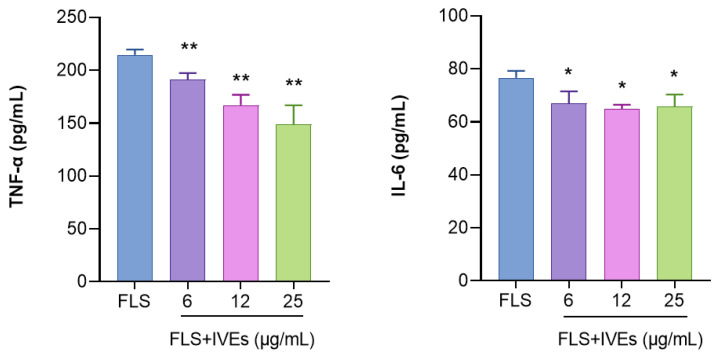
Effects of *Illicium verum* extracts on inflammatory factors in RA-FLS. The levels of TNF-α and IL-6 in RA-FLS were measured by using ELISAs. The data are expressed as means ± SDs (*n* = 4). * and ** represent *p* < 0.05 and 0.01 compared with the untreated group, respectively.

We measured the production of TNF-α and IL-6 in RA-FLS in order to determine the anti-inflammatory activity of IVEs. Incubation of RA-FLS with IVEs led to a reduction in the release of TNF-α and IL-6 in a concentration-dependent manner (Figure 3).

### 3.4. IVEs Regulated the Cell Metabolomic Profiles of RA-FLS

#### 3.4.1. Multivariate Statistical Analysis

**Figure 4:**
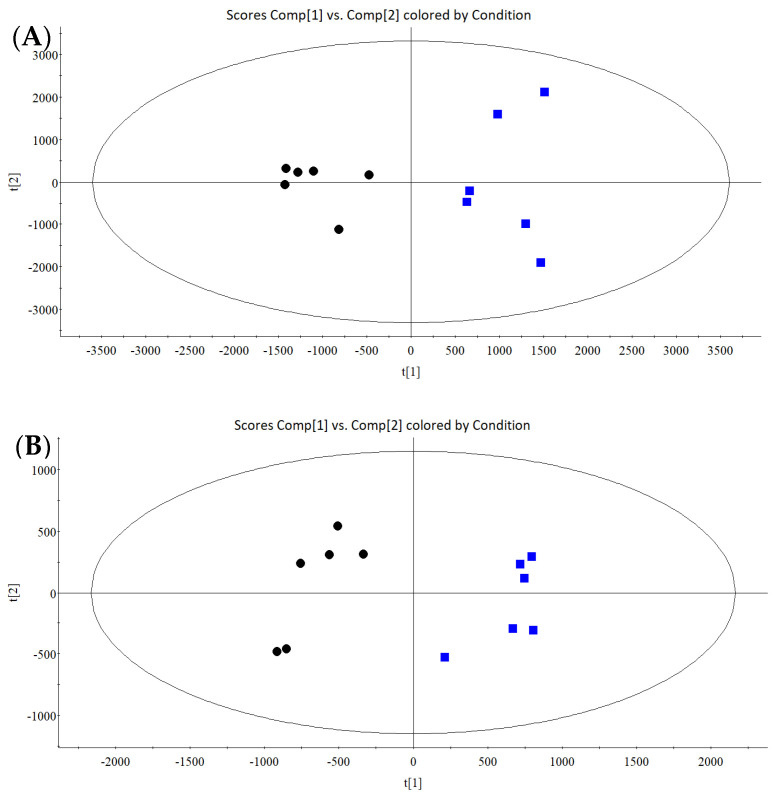
OPLS-DA score plots of the untreated and the IVE-treated groups in the positive (**A**) and negative (**B**) modes. The black dots refer to the untreated group and the blue square refers to the IVEs-treated group; *n* = 6 per group.

UPLC-HDMS analysis produced the cell metabolomic profiles in both the positive- and negative-ion modes (Figure S1). Multivariate statistical analysis was then performed to reveal the cellular metabolite changes after IVEs administration. PCA (Figure S2) and OPLS-DA (Figure 4) were used to locate the differentiating variables in order to assess the intrinsic differences between the treated and untreated groups (Figure 4) and showed that the metabolic network within the RA-FLS cell line was affected by IVEs.

#### 3.4.2. Differential Metabolite Identification

**Figure 5:**
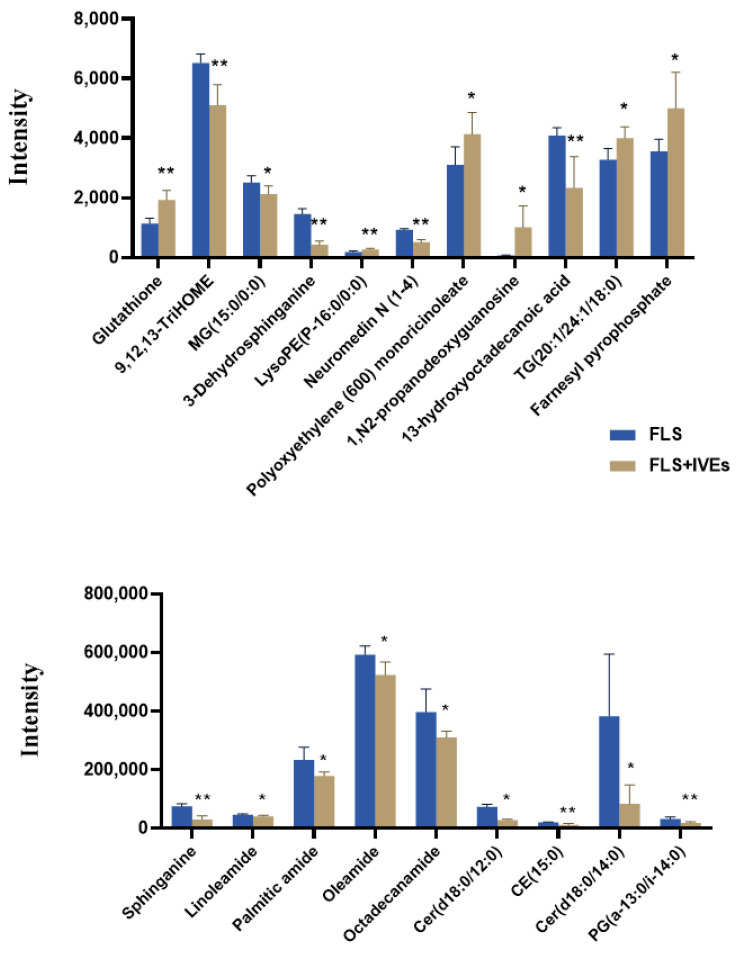
The changes in the relative peak intensities of 20 identified differential metabolites. The data are expressed as means ± SDs (*n* = 6). * and ** represent *p* < 0.05 and 0.01 compared with the untreated group, respectively.

**Figure 6:**
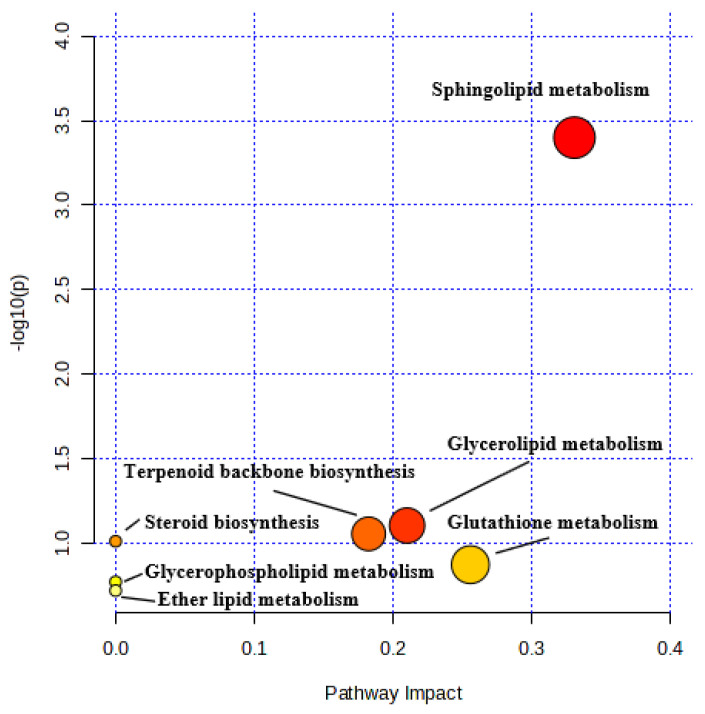
Pathway enrichment analysis diagram depicting the altered metabolic pathways in RA-FLS from the untreated- and IVE-treated groups.

Using the VIP and the s-plot diagrams (VIP > 1, *p* < 0.05), twenty features were identified as significantly differential metabolites (Table 1 and Figure 5). Compared with the untreated group, six metabolites were up-regulated, significantly contributing to the effects of IVEs. These included glutathione, lysoPE (P-16:0/0:0), polyoxyethylene monoricinoleate, 1,N2-propanodeoxyguanosine, TG (20:1/24:1/18:0), and farnesyl pyrophosphate. Fourteen metabolites were significantly down-regulated after IVEs treatment and these included 9,12,13-TriHOME, MG(15:0/0:0), sphinganine, 3-dehydrosphinganine, linoleamide, palmitic amide, oleamide, octadecanamide, neuromedin N(1-4), Cer (d18:0/12:0), CE (15:0), 13-hydroxyoctadecanoic acid, Cer (d18:0/14:0), and PG (a-13:0/i-14:0). As indicated in Figure 6, these metabolites were involved in seven metabolic pathways, including sphingolipid, glycerophospholipid, glycerolipid, glutathione, and ether lipid metabolism as well as terpenoid backbone and steroid biosynthesis.

## 4. Discussion

It has been reported that are increased levels of cholesterol and lipoproteins in the synovial fluid of RA patients, and their synovial tissues are similar to those of solid tumors, existing in a hypoxic and cytokine-rich microenvironment. However, long-activated RA-FLS exhibit tumor-like proliferation, invasion, and migration characteristics, and related studies have shown abnormal lipid metabolism in fibroblastic synovial cells of RA [19,20,21]. Lipid metabolism plays a crucial role in cellular metabolism and the regulation of immune responses [2]. The majority of lipids found in eukaryotes require at least four metabolic networks, including sphingolipids, glycerophospholipids, glycerolipids, and non-esterified fatty acids [22]. These produce a variety of intermediates that are necessary for maintaining cellular homeostasis and regulating physiological functions in organisms [23]. Both excessive lipid accumulation and deficiencies in fatty acid oxidation are linked to increased lipotoxicity, which directly contributes to the progression of fibrosis in RA patients [24]. It has been found that the serum of RA patients have high levels of lipid peroxidation products and oxidative stress, which are closely related to the inflammatory response, free radical metabolism, and immune cell activity of the disease [25].

Sphingolipids constitute a primary category of highly bioactive lipids, possessing the capability to activate distinct protein targets, encompassing lipases, phosphatases, and kinases. Preliminary evidence suggests that these might be relevant in the pathophysiology of RA [26,27]. Sphingolipids and their metabolites, such as ceramide, sphingosine, and sphingosine-1-phosphate, are implicated in a wide array of cellular processes encompassing differentiation, cellular senescence, apoptosis, and proliferation [28]. Sphingosine, being one of the principal sphingolipid bases, forms the fundamental framework of all sphingolipids, and they provide these molecules with their unique characteristics [29].

Ceramides are present at relatively low concentrations and they act as indicators of lipid surplus, triggering various cellular stress responses and ultimately causing apoptosis, and are produced from sphingomyelins in the cell membrane after IL-1β/TNF-α receptor activation [30,31,32]. The ceramide levels have been observed to be markedly elevated in the livers and muscles of RA model mice [33]. A wide range of sphingolipids species, as well as their precursors and intermediate metabolites, were found in human articular synovial fluid [34]. The levels of certain sphingolipids were significantly elevated in the serum samples of RA patients in contrast to the healthy control group [35]. Our study found a significant reduction in the levels of several sphingolipids metabolites in RA-FLS, indicating a trend towards normalization after the addition of IVEs. These results imply that the effect of IVEs on ameliorating RA is closely associated with the regulation of the sphingolipid metabolism.

There is increasing evidence that lipoproteins change during the development of RA, and blood lipids, including triglycerides and cholesterol, have an effect on circulating immune cells [36]. Reactive oxygen species (ROS) primarily target lipids, initiating lipid peroxidation that produces mutagenic by-products. However, a dysregulated glycerophospholipid metabolism is linked to increased systemic inflammation, altered neutrophil activation signaling, and the production of proteolytic enzymes and ROS, all of which contribute to cartilage tissue degradation [37,38]. In this study, the levels of MGs and PGs were significantly decreased after IVEs treatment, while the level of LysoPEs were markedly increased, indicating that the metabolism of glycerophospholipids in RA-FLS could be regulated by IVEs.

Glutathione regulates immune responses to maintain homeostasis and reduces inflammation, with implications for autoimmune disorders [39]. It is metabolized to protect cells from oxidative stress, detoxify radicals and peroxides, conjugate with electrophilic compounds, and defend against ROS and toxic products [40]. In our study, the glutathione levels were elevated in RA-FLS treated with IVEs, when compared to controls. This suggests that IVEs may influence the glutathione metabolism.

## 5. Conclusions

This study demonstrates that IVEs can effectively suppress the proliferation and migration of RA-FLS, as well as their ability to express inflammatory factors. In addition, our findings suggest that the treatment of RA-FLS with IVEs had a regulatory effect on the metabolic processes involving lipids and the amino acid metabolism. This indicates that IVEs may be able to reduce the inflammation observed in RA patients, thereby potentially offering a new avenue for therapeutic intervention in managing this condition. *I. verum*, a medicinal plant with a history in traditional medicine, may provide an affordable treatment for RA with relatively few side effects. Although *I. verum* holds significant promise as a potential therapeutic agent for RA, further research is essential to address the limitations and challenges associated with its use. Comprehensive clinical trials are needed to determine its efficacy, optimal dosing, and potential side effects, as well as to establish standardized protocols for its integration into current RA treatment regimens for maximal benefits to RA patients.

## Figures and Tables

**Table 1:** The detailed list of metabolites that were significantly increased or decreased in RA-FLS associated with IVEs treatment.

No	Metabolite	Formula	Retention Time (min)	*m*/*z*	Adducts	Trends	Category
1	Glutathione	C_10_H_17_N_3_O_6_S	0.91	308.0917	M + H	↑**	Amino acids and peptides
2	9,12,13-TriHOME	C_18_H_34_O_5_	2.65	329.2332	M − H	↓**	Fatty Acyls
3	MG (15:0/0:0)	C_18_H_36_O_4_	3.93	315.2535	M − H	↓*	Glycerophospholipids
4	Sphinganine	C_18_H_39_NO_2_	4.00	302.3059	M + H	↓**	Sphingolipids
5	3-Dehydrosphinganine	C_18_H_37_NO_2_	4.27	300.2903	M + H	↓**	Sphingolipids
6	LysoPE (P-16:0/0:0)	C_21_H_44_NO_6_P	4.85	436.2807	M − H	↑**	Glycerophospholipids
7	Linoleamide	C_18_H_33_NO	5.89	280.2646	M + H	↓*	Fatty acyls
8	Palmitic amide	C_16_H_33_NO	6.64	256.2648	M + H	↓*	Fatty acyls
9	Oleamide	C_18_H_35_NO	6.89	282.2804	M + H	↓*	Fatty acyls
10	Octadecanamide	C_18_H_37_NO	8.10	284.296	M + H	↓*	Amino acids, peptides, and analogs
11	Neuromedin N (1-4)	C_26_H_40_N_4_O_6_	8.14	1007.5872	2M − H	↓**	Fatty acyls
12	Polyoxyethylene (600) monoricinoleic acid	C_21_H_40_O_3_	8.58	341.3032	M + H	↑*	Fatty acyls
13	1,N2-propanodeoxyguanosine	C_13_H_17_N_5_O_4_	9.31	330.1163	M + Na	↑*	Nucleoside and nucleotide analogs
14	Cer (d18:0/12:0)	C_30_H_61_NO_3_	9.56	484.4722	M + H	↓*	Sphingolipids
15	CE (15:0)	C_42_H_74_O_2_	10.50	609.5558	M − H	↓**	Sterol lipids
16	13-hydroxyoctadecanoic acid	C_18_H_36_O_3_	10.53	599.5258	2M − H	↓**	Fatty acyls
17	Cer (d18:0/14:0)	C_32_H_65_NO_3_	10.62	512.504	M + H	↓*	Sphingolipids
18	PG (a-13:0/i-14:0)	C_33_H_65_O_10_P	11.07	653.4431	M + H	↓**	Glycerophospholipids
19	TG (20:1/24:1/18:0)	C_65_H_124_O_5_	11.54	1007.9286	M + Na	↑*	Glycerolipids
20	Farnesyl pyrophosphate	C_15_H_28_O_7_P_2_	11.86	383.1421	M + H	↑*	Terpenoid backbone biosynthesis

Note: ↑ means that the level of the corresponding metabolite is increasing in IVEs-treated group compared to the untreated group; ↓ means that the level of the metabolite is decreasing in the IVEs-treated group compared to the untreated group. * *p* < 0.05; ** *p* < 0.01.

## Data Availability

Data are contained within the article and Supplementary Materials.

## Supplementary Materials

The following supporting information can be downloaded at: https://www.mdpi.com/article/10.3390/metabo14100517/s1, Figure S1: UPLC-HDMS chromatograms in positive mode and negative mode; Figure S2: Score plots of cell metabolic footprint PCA analysis of RA-FLS in positive mode and negative mode.
