# Supplementary material for: Ultra-High-Performance Liquid Chromatography–High-Definition Mass Spectrometry-Based Metabolomics to Reveal the Potential Anti-Arthritic Effects of Illicium verum in Cultured Fibroblast-like Synoviocytes Derived from Rheumatoid Arthritis
Source: Metabolites. 2024 Sep 25;14(10):517. doi: 10.3390/metabo14100517 (PMC11509614; doi:10.3390/metabo14100517)

Supplementary Materials:

Figure S1. UPLC-HDMS chromatograms in positive mode and negative mode. (A) BPI chromatogram of cell metabolism footprint sample in positive mode. (B) BPI chromatogram of cell metabolism footprint sample in negative mode.

(A)

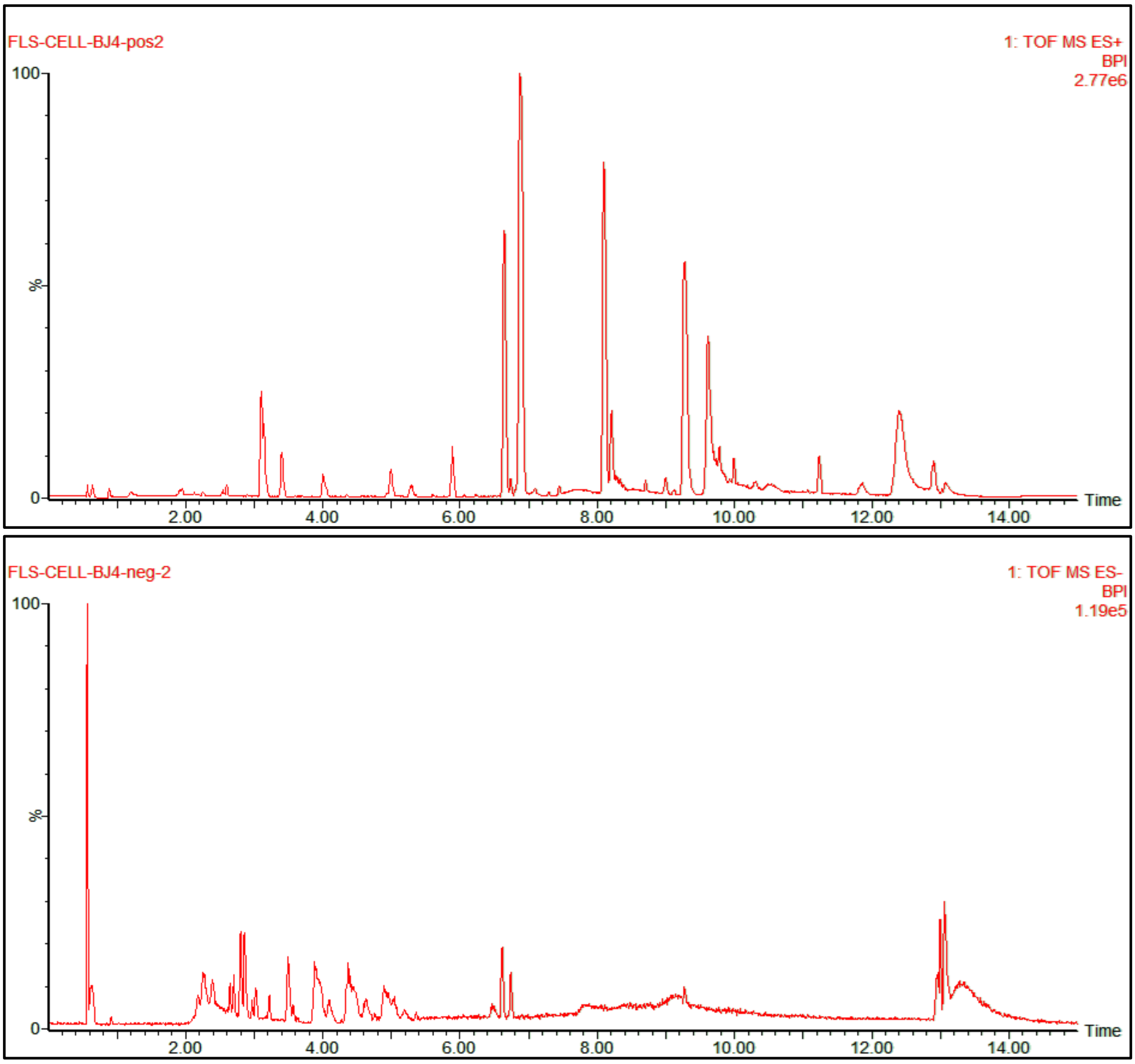

(B)

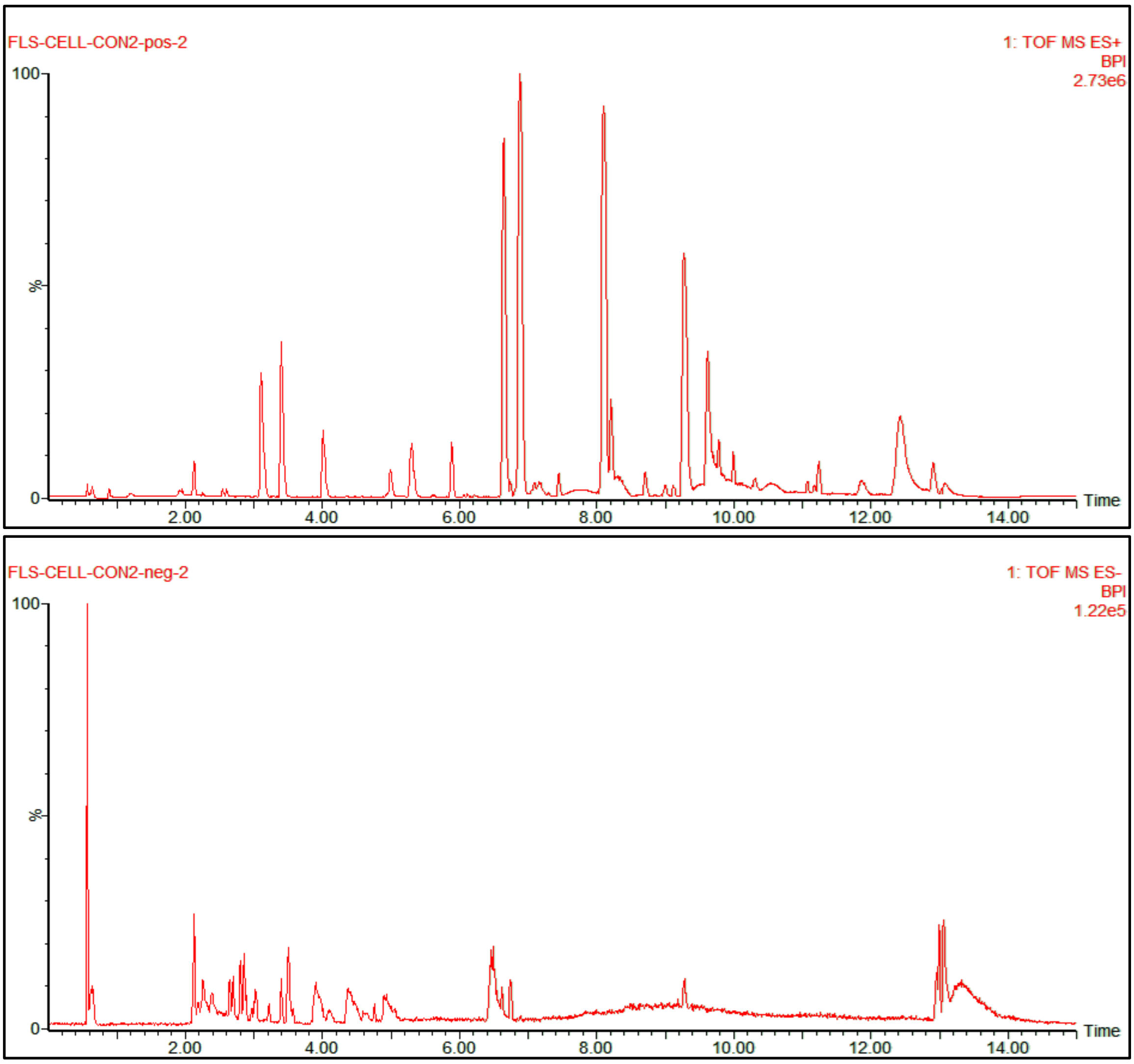

FLS-CELL-CON2-pos-2
1: TOF MS ES+
BPI
2.73e6
100
%
0
2.00
4.00
6.00
8.00
10.00
12.00
14.00
Time
FLS-CELL-CON2-neg-2
1: TOF MS ES-
BPI
1.22e5
100
%
0
2.00
4.00
6.00
8.00
10.00
12.00
14.00
Time

Figure S2. Score plots of cell metabolic footprint PCA analysis of RA-FLS in positive mode and negative mode. The black dots refer to untreated group and the blue dots refer to IVEs-treated group; n = 6 per group.

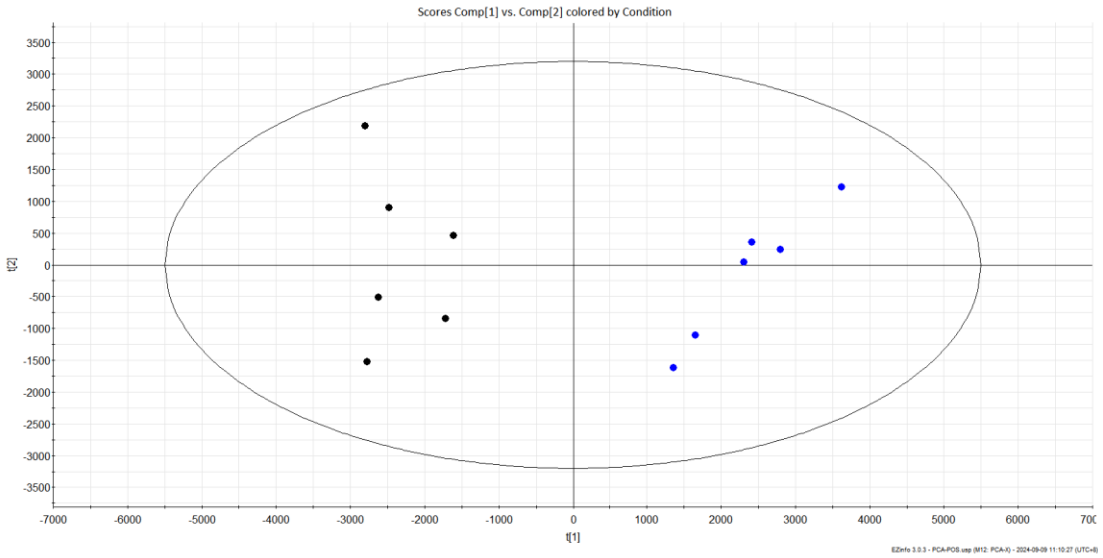

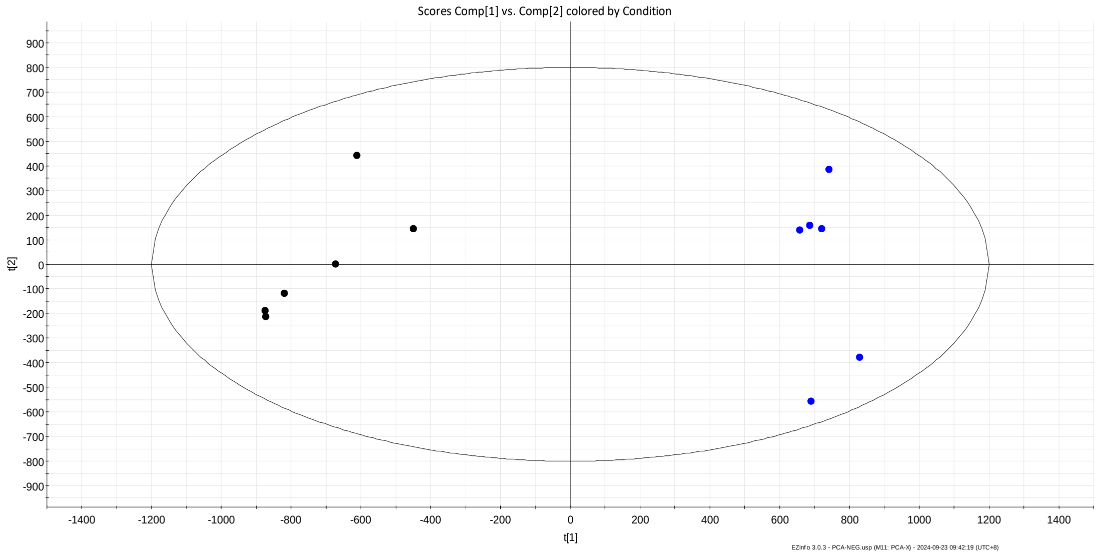

Supplement: Supplementary file 1 [file metabolites-14-00517-s001.zip › metabolites-3223033-supplementary.pdf]
